# Analysis of Intestinal Microbiota Differences and Functional Prediction Between Sichuan-Tibetan Black Pigs and Landrace Pigs

**DOI:** 10.3390/microorganisms14010258

**Published:** 2026-01-22

**Authors:** Lichun Jiang, Yi Qing, Kaiyuan Huang, Huiling Huang, Chengmin Li, Yanci Li

**Affiliations:** 1 School of Biological and Pharmaceutical Sciences, Mianyang Teachers’ College, Mianyang 621000, China; 17760556621@163.com (K.H.); 18380527400@163.com (C.L.); 15388466279@163.com (Y.L.); 2 School of Life Science, Mianyang Teachers’ College, Mianyang 621000, China; qyyq0924@163.com (Y.Q.); m18227952859@163.com (H.H.)

**Keywords:** Sichuan-Tibet black pigs, landrace pigs, intestinal microbiota, structure, function prediction

## Abstract

This study aimed to investigate the structural differences and functional potential of the gut microbiota between Sichuan-Tibetan black pigs (*n* = 5) and Landrace pigs (*n* = 5) under identical rearing conditions. Fecal samples were collected and subjected to 16S rRNA gene sequencing followed by comprehensive bioinformatics analysis. The results revealed 963 and 910 operational taxonomic units (OTUs) in Sichuan-Tibetan black pigs and Landrace pigs, respectively, with 808 OTUs shared between the two breeds. While both breeds shared Firmicutes, Bacteroidota, and Proteobacteria as the dominant phyla, significant compositional differences were observed at the genus level. Sichuan-Tibetan black pigs exhibited higher abundance of *Escherichia-Shigella*, *Streptococcus*, *Prevotella*, *Parabacteroides*, and *Collinsella*, whereas Landrace pigs were enriched in *Bacteroides*. Alpha diversity analysis showed no significant differences in Shannon, Simpson, or ACE indices, though the Chao index differed markedly between the two groups. Beta diversity analysis (PCoA and NMDS) confirmed distinct microbial community structures between the breeds. Functional prediction analysis demonstrated that metabolic pathways dominated in both groups, but with notable functional differentiation: the microbiota of Sichuan-Tibetan black pigs showed significant enrichment in biosynthesis of secondary metabolites, microbial metabolism in diverse environments, and amino acid biosynthesis; whereas, Landrace pigs were characterized by enhanced carbon and energy metabolism pathways. Additionally, BugBase phenotype prediction revealed significant differences in stress tolerance, cell wall properties, and oxygen utilization capabilities between the two groups. These findings provide valuable insights into the breed-specific characteristics of gut microbiota in swine and establish a foundation for further research on host-microbe interactions and their implications for animal health and nutrition.

## 1. Introduction

The porcine intestinal ecosystem comprises the intestinal mucosa, gut microbiota, and associated immune tissues, which have co-evolved with the host to form an integrated physiological system [1]. Substantial evidence indicates that the gut microbiota plays pivotal roles in maintaining host health and supporting growth performance [2,3]. Since the prohibition of antibiotics in swine production, research on porcine gut microbiota has gained increasing attention as a promising area for developing alternative strategies [4]. The porcine gut harbors a diverse microbial community dominated by Firmicutes, Bacteroidetes, Proteobacteria, and Actinobacteria [5]. Among these, Firmicutes and Bacteroidetes constitute the predominant phyla and are crucial for intestinal homeostasis and nutrient metabolism. Firmicutes contribute to host nutrition through the production of various digestive enzymes that facilitate nutrient absorption from feed, while also inhibiting pathogenic bacteria, modulating intestinal immunity, and enhancing overall disease resistance [6]. Bacteroidetes possess potent polysaccharide-degrading capabilities, converting indigestible dietary fibers into absorbable monosaccharides that provide additional energy sources, and participate in bile acid metabolism that influences lipid digestion and absorption in pigs.

Host genetics, particularly breed differences, significantly shape the gut microbiota composition in animals. Cheng et al. [7] demonstrated that different pig breeds exhibit distinct gut microbiota structures, with Pietrain pigs showing significant variations in Firmicutes and Bacteroidetes compared to three other breeds, confirming breed-specific microbial patterns. Ma et al. [8] revealed substantial differences in microbial diversity, probiotic abundance, and predicted metabolic functions between wild boars and fourth-generation hybrid wild pigs through a comparative analysis of gut microbiota and metabolites. Similarly, Lu et al. [9] reported breed-specific variations in dominant and specialized microbial species in the ileum, suggesting associations with differences in intestinal physiological functions among pig breeds.

Despite the commercial importance of Tibetan black pigs and Landrace pigs in the domestic pork market, comparative studies on their gut microbiota remain limited. Comparing these two pure breeds is scientifically rational: both have stable genetic backgrounds (Tibetan black pigs are indigenous lines adapted to the Tibetan Plateau’s high-altitude and cold environment; Landrace pigs are lean-type lines with global unified breeding standards), which eliminates hybrid genetic heterozygosity interference to accurately identify genetics-driven microbial traits. Meanwhile, their distinct phenotypes (Tibetan black pigs: strong stress resistance, slow growth, excellent meat quality; Landrace pigs: fast growth, high feed conversion efficiency, high lean meat rate) provide an excellent model to explore microbiota’s role in mediating host environmental adaptation and production performance. Landrace pigs were selected over other breeds for their strong commercial representativeness as core parental lines in global hybrid systems (e.g., Duroc × (Landrace × Yorkshire)), high genetic/phenotypic uniformity reducing intra-breed microbial variation, and solid research foundation facilitating result interpretation and verification.

This study aims to address this knowledge gap by employing metagenomic sequencing to investigate the structural and functional differences in gut microbiota between these two breeds. Specifically, we seek to compare the community structure and diversity of gut microbiota, identify breed-specific microbial signatures, and elucidate differences in functional metabolic pathways. Our findings will provide theoretical foundations for understanding host–microbe interactions in different pig breeds and support future research on gut microbiota functions in Tibetan black pigs, Landrace pigs, and related species.

## 2. Materials and Methods

### 2.1. Sample Collection in Animals

This study selected 5 healthy Sichuan-Tibetan black pigs (zz) and 5 healthy Landrace pigs (cf) from the Jiangyou pig farm in Mianyang. The selected pigs had consistent bloodline purity, as purebred individuals, and complete ancestry records. All pigs were of the same parity, with similar body weight and no history of disease or antibiotic treatment. The animals were raised under the same feeding and management conditions. They were housed in standard pens with a controlled environment, including stable temperature and humidity. The feeding diet was a uniform commercial corn-soybean meal ration that meets the nutritional requirements of growing pigs. All pigs had free access to feed and clean drinking water throughout the rearing period. Fresh feces, approximately 200 g per sample, were collected from the center of the fecal heap immediately after the pigs defecated. The collected samples were rapidly frozen in liquid nitrogen to preserve microbial activity and then stored at −20 °C for subsequent experiments.

### 2.2. Genome Extraction and Illumina Miseq Sequencing

DNA assay kit (E.Z-N.A.)^®^ (Omega Bio-Tek Inc., Norcross, GA, USA) was used. Total DNA was extracted from two sets of fecal samples using a soil DNA kit. After extraction, the DNA concentration and purity were detected using a NanoDrop spectrophotometer (Thermo Fisher Scientific Inc., Waltham, MA, USA), and precise quantification was performed using a Qubit fluorescence quantitative analyzer (Thermo Fisher Scientific Inc., Waltham, MA, USA); DNA integrity was evaluated by 1% agarose gel electrophoresis. Three technical replicates were set up for each sample to ensure the reliability of the results. Using 10 ng fecal DNA as a PCR amplification template, the V3–V4 regions of 16S rDNA were amplified using a PCR kit (Trans Gen AP221-02, TransGen Biotech Co., Ltd., Beijing, China). The specific primers 338F (ACTCCTACGGGAGGCAGCAG) and 806R (GGACTACHVG GGTWTCTAAT) were selected, and the amplification system was set to 20 μL. The amplification process is set as follows: pre denaturation at 95 °C (3 min); 95 °C denaturation (30 s), 55 °C annealing (30 s); extend at 72 °C (45 s), after 27 cycles, further extend at 72 °C for 10 min. The purity of PCR amplification fragment was detected by agarose gel electrophoresis and purified by gel recovery kit. The gel was then cut by agarose gel electrophoresis to recover the target fragments, and were finally mixed in proportion. Paired-end library was constructed using a library construction kit and sequence on an Illumina MiSeq platform (Illumina, San Diego, CA, USA). The library construction and sequencing were completed by Shanghai Meiji Biomedical Technology Co., Ltd. (Shanghai, China). The sequencing data was uploaded to the Meiji platform for data analysis.

### 2.3. Bioinformatics Analysis

The initial quality control of raw sequencing data was performed with FASTP. The QIIME 2 version 2022.8 pipeline [10] was then employed to filter high-quality reads. Utilizing the UPARSE algorithm [11], valid sequences were clustered into operational taxonomic units (OTUs) based on a 97% similarity threshold, followed by their taxonomic classification against the SILVA 138.1 database [12]. Sequencing depth adequacy was confirmed via rarefaction curves. Pan-genome and core-genome analyses were conducted to delineate both the core gene set shared by all strains and the total pan-genome within the species. To evaluate microbial community richness, alpha diversity indices (Shannon, Observed species, and ACE) were determined with QIIME. Structural differences among groups were examined through beta diversity analyses, specifically Principal Coordinate Analysis (PCoA) and Non-metric Multidimensional Scaling (NMDS). For species composition analysis, species Venn diagrams were constructed to identify core microorganisms; community composition was characterized; in addition, Circos plots depicting the associations between samples and species were generated, and the corresponding sample-species relationships were elucidated. Finally, to characterize functional shifts in the metabolic pathways between the two microbial groups, phenotypic profiles were predicted utilizing BugBase. Subsequently, functional annotation of the communities was performed employing the FAPROTAX database, a tool extensively applied in microbial diversity studies.

## 3. Results

### 3.1. Sequencing Depth and Sample Information

**Figure 1:**
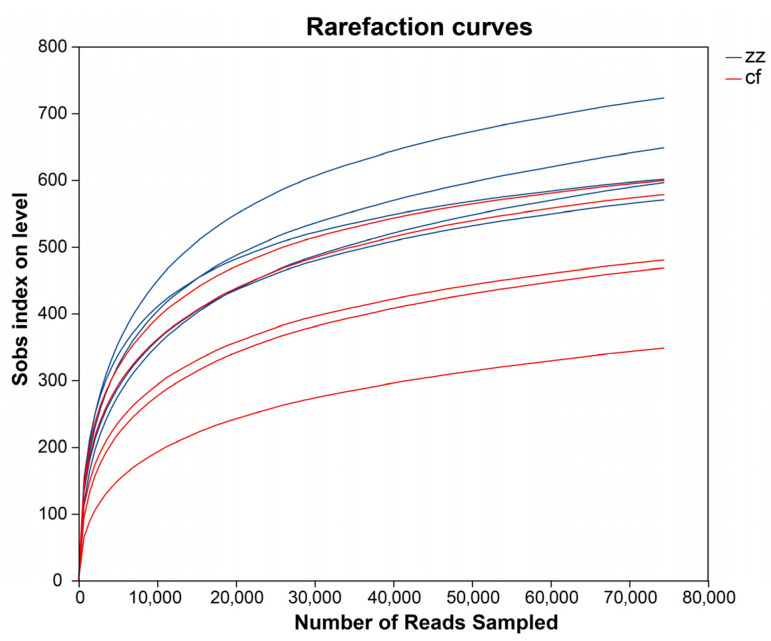
Analysis of Shannon index dilution curve.

To assess the sufficiency of the sequencing data, microbial alpha diversity indices and Sobs rarefaction curves were generated for both samples from Sichuan-Tibetan black pigs and Landrace pigs [13]. Sequencing data were normalized to a depth of 45,000 reads per sample to ensure consistency across groups. As shown in Figure 1, the Sobs rarefaction curves plateau, approaching a stable asymptote. This indicates that the sequencing depth and coverage were adequate for subsequent analytical stages.

**Figure 2:**
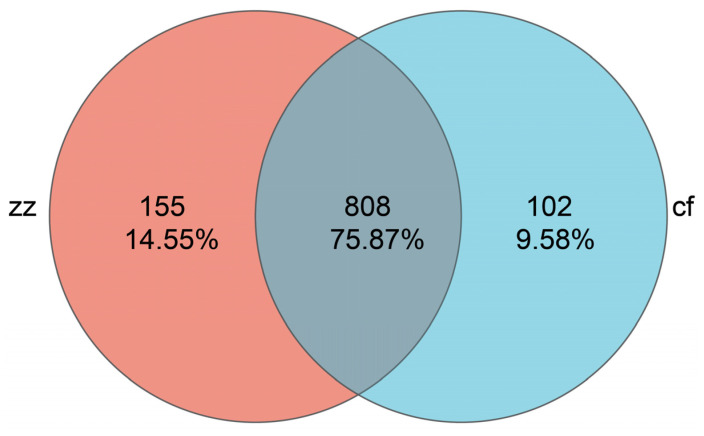
Venn map of inter-group species.

Based on OTU clustering at a 97% similarity threshold, a Venn diagram was constructed to quantify shared and unique OTUs between the two groups. Statistical analysis revealed a total of 808 species across the populations, with Sichuan-Tibetan black pigs and Landrace pigs harboring 155 and 102 unique OTUs, respectively, as depicted in Figure 2.

**Figure 3:**
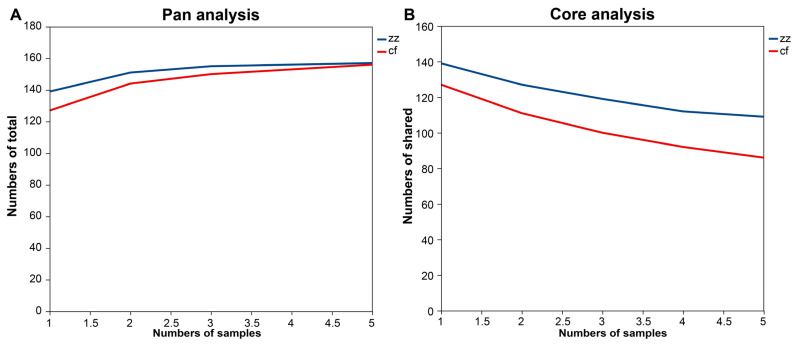
Pan (**A**)/Core (**B**) analysis curve.

Furthermore, based on the sequencing data, Pan and Core species analysis was conducted at the genus taxonomic level. From the Pan curve, it was observed that with the increase in the number of samples, the number of bacterial genera in both Sichuan-Tibetan black pigs and Landrace pigs gradually increased and approached the same value (Figure 3A). From the Core curve, it could be seen that as the number of intestinal flora samples of Tibetan black pigs and Landrace pigs increased and the number of shared bacterial genera among each sample of their intestinal flora gradually decreased (Figure 3B). These two curves indicate that at the genus taxonomic level, the number of bacterial genera in the intestinal flora of Sichuan-Tibetan black pigs and the number of shared bacterial genera among each sample are higher than those in Landrace pigs. However, with the increase in the number of samples, the difference in the total number of bacterial genera between the two breeds decreases, while the number of shared bacterial genera among individual samples of both breeds gradually decreases, and the difference in the number of shared bacterial genera becomes more pronounced.

### 3.2. Diversity Analysis of Gut Microbiota

**Figure 4:**
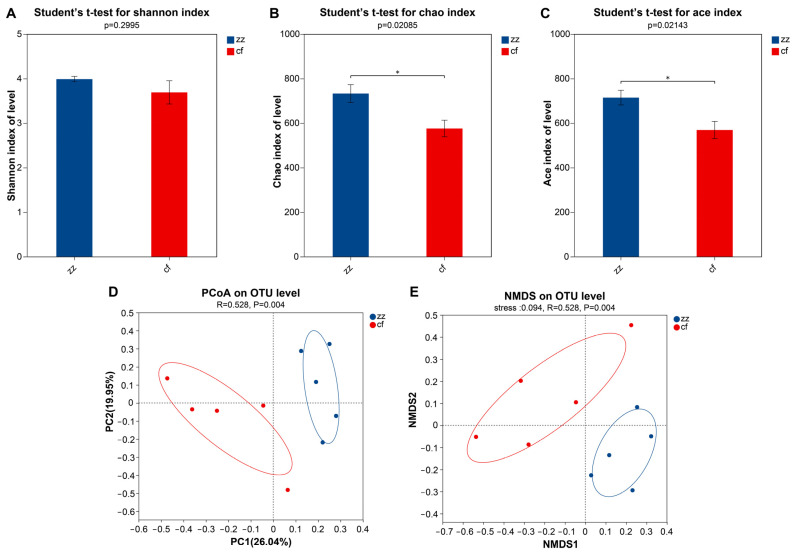
Diversity analysis of the gut microbiota. (**A**–**C**) Alpha diversity analysis of the Chao, Shannon, and Ace indices. (**D**) Principal coordinates analysis (PCoA). (**E**) Non-metric multi-dimensional scaling (NMDS). The asterisk (*) above the bars in panels (**B**,**C**) indicates a statistically significant difference between the two groups (*p* < 0.05), as determined by Student’s *t*-test.

To evaluate the changes in gut microbiota of Sichuan-Tibetan black pigs and Landrace pigs, we used richness (Chao and Ace) and evenness (Shannon) indices to assess alpha diversity. Statistical analysis was performed using Student’s t-test to compare differences in alpha diversity indices between the two breeds, with significance defined as *p* < 0.05. As shown in Figure 4, compared with the cf group, the zz group exhibited higher microbial evenness, which is reflected in the increase in Shannon index (*p* = 0.2995, not significant). In addition, as shown by the Chao index, the microbial richness of the cf group significantly increased (*p* = 0.02085). We further investigated the overall structural changes in microbial communities using Principal Coordinate Analysis (PCoA) based on Bray–Curtis distance and Non Metric Multidimensional Scaling (NMDS) [14,15]. PCoA revealed a clear separation between the two breeds (Figure 4D), with PC1 and PC2 explaining 26.04% and 19.95% of the total variance, respectively. Permutational multivariate analysis of variance (PERMANOVA) confirmed significant differences in microbial community structure (R = 0.528, *p* = 0.004). Similarly, NMDS (stress = 0.094) confirmed substantial differences in the composition and structure of the bacterial communities between the two breeds (Figure 4E), with PERMANOVA results consistent with PCoA (R = 0.528, *p* = 0.004).

### 3.3. Species Composition Analysis

**Figure 5:**
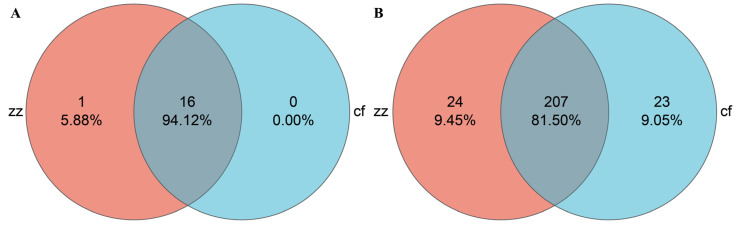
Venn diagrams at the phylum level (**A**) and genus level (**B**).

**Figure 6:**
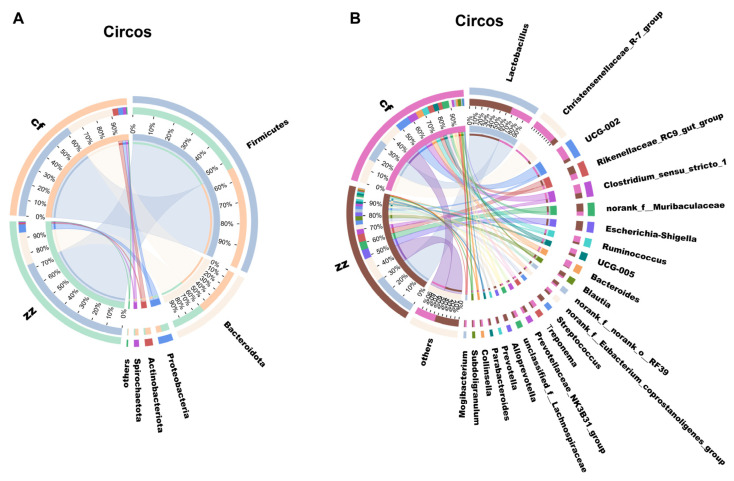
Circos plots at the phylum level (**A**) and genus level (**B**).

Taxonomic analysis was conducted on the characteristic sequences of Sichuan-Tibetan black pigs and Landrace pigs, resulting in 17 phyla, 25 classes, 58 orders, 99 families, 253 genera, and 457 species. At the phylum classification level, a total of 17 phyla were detected in the gut microbiota of Sichuan-Tibetan black pigs and Landracei pigs, including 16 core phyla, 1 phylum unique to Sichuan-Tibetan black pigs, and no phylum unique to Landrace pigs (Figure 5). The dominant phyla of gut microbiota in Sichuan-Tibetan black pigs and Landrace pigs (Figure 6A) are Firmicutes, Bacteroidetes, and Proteobacteria. The abundance of Firmicutes (73%) in the gut microbiota of Sichuan-Tibetan black pigs is higher than that of Landrace pigs (62%), Bacteroidetes (20%) is lower than that of Landrace pigs (29%), and the abundance of other phyla is not significantly different.

At the taxonomic level, a total of 254 genera were detected, including 24 genera unique to Sichuan-Tibetan black pigs, 23 genera unique to Landrace pigs (Figure 6A), and 23 dominant bacterial genera (Figure 6B). The dominant bacterial genera in the gut microbiota of Sichuan-Tibetan black pigs were *Lactobacillus* (31%), *Rikenellaceae_RC9_gut_group* (6%), *Escherichia Shigella* (7%), and *norank_f__Muribaculaceae* (6%); The dominant bacterial genera in the gut microbiota of Landrace pigs are *Lactobacillus* (16%), Kristensen’s bacteria (17%), *Ruminococcus* (10%), *Clostridium sensu_stricto_1* (6%), and *Bacteroides* (5%). It should be clarified that *Escherichia-Shigella*, *Streptococcus*, *Prevotella*, *Parabacteroides*, and *Collinsella* are not absent in Landrace pigs but are present at significantly lower abundances compared to Sichuan-Tibetan black pigs (defined as “enriched” in Sichuan-Tibetan black pigs). Similarly, Bacteroides is not unique to Landrace pigs but shows higher enrichment in this breed. The relatively low abundance of *Escherichia-Shigella*, *Streptococcus*, and *Prevotella* in Landrace pigs may be related to their genetic background (selected for fast growth and lean meat rate) and metabolic characteristics, as these genera are often associated with fiber degradation and environmental adaptation—traits more prominent in Sichuan-Tibetan black pigs. Additionally, differences in gut pH and nutrient availability between the two breeds may contribute to the variation in these genera. Regarding the individual sample CF5 (a Landrace pig), the observed variation in its microbial composition may be attributed to individual differences in gut microenvironment (e.g., pH, digestive enzyme activity) or minor variations in feed intake, even under uniform rearing conditions. Such individual variation is common in microbial community studies.

**Figure 7:**
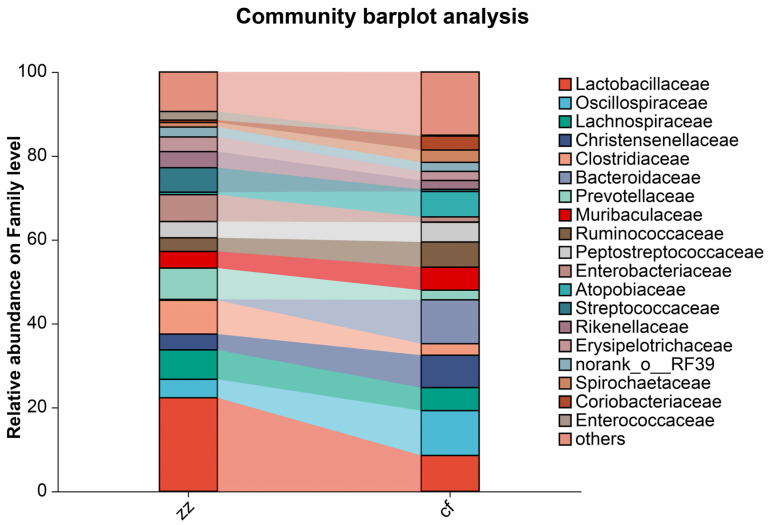
Stacked bar chart of species composition.

Analysis of the gut microbiota composition at the family level across different developmental stages; (Figure 7) identified the top five dominant bacterial families in Sichuan-Tibetan black pigs as *Lactobacillaceae* (22.31%), unclassified taxa (9.42%), *Clostridiaceae* (8.08%), *Prevotellaceae* (7.48%), and *Lachnospiraceae* (7%). The top five dominant bacterial species in the gut microbiota of Landrace pigs are others (15.09%), *Oscillospiraceae* (10.70%), *Bacteroidaceae* (10.47%), *Lactobacillaceae* (8.54%) and *Christensenellaceae* (7.74%). Within the intestinal microbiome of Sichuan-Tibetan black pigs, the abundance of Lactobacillus is significantly higher than that of Landrace pigs. In the gut microbiota of Landrace pigs, the abundance of *Oscillospiraceae* and *Bacteroidaceae* bacteria is higher than that of Sichuan-Tibetan black pigs.

### 3.4. Functional Prediction of Intestinal Flora in Chuanzang Black Pigs and Landrace Pigs

**Figure 8:**
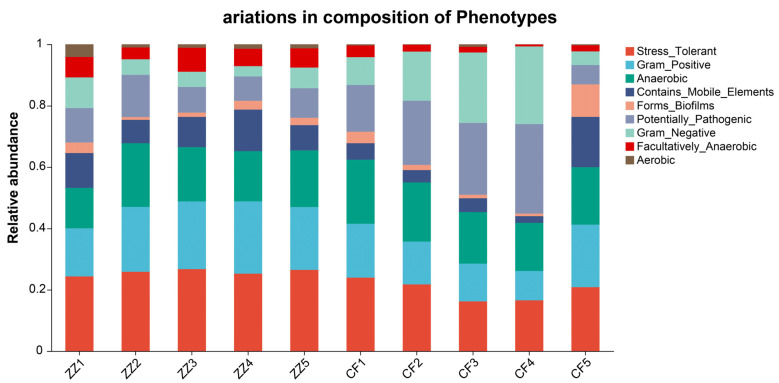
Phenotype prediction results.

**Figure 9:**
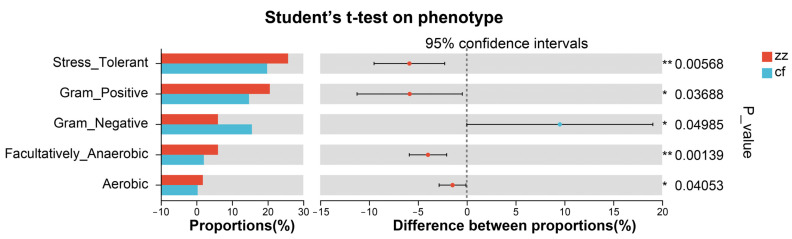
Results of phenotype group difference test. * denotes a significant difference (0.01 ≤ *p* < 0.05), and ** denotes a highly significant difference (*p* < 0.01).

Through BugBace phenotype prediction, the results showed that there were significant differences (*p* < 0.05) within the enteric microbial community of Sichuan-Tibetan black pigs and Landrace pigs in five phenotypes: pressure tolerance, Gram positive, Gram negative, biological environment adaptability, and oxygen utilization efficiency. The gut microbiota of Sichuan-Tibetan black pigs was higher than that of Landrace pigs in all phenotypes expect Gram Negative and Pathogenic (Figure 8 and Figure 9).

**Figure 10:**
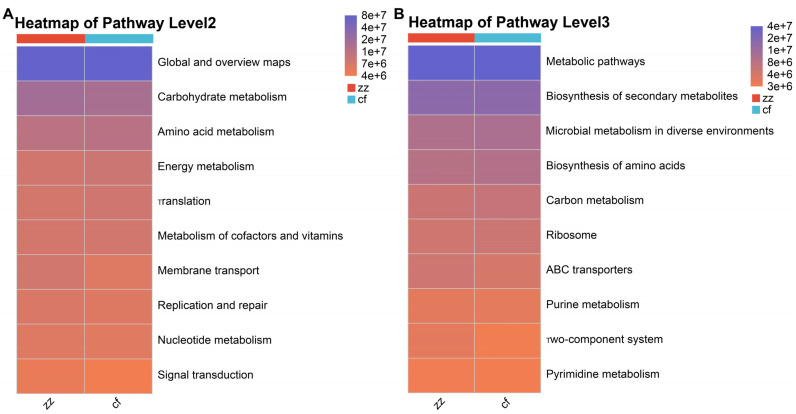
Functional prediction of KEGG pathway at the second level (**A**) and third level (**B**).

PICRUSt2 was employed to predict the functional profiles of gut microbiota from Sichuan-Tibetan black pigs and Landrace pigs based on sequencing data, in conjunction with the KEGG database. The results revealed that metabolic functions dominated the primary functional categories of the gut microbiota in both pig breeds, followed by genetic information processing, environmental information processing, and cellular processes. This suggests that the gut microbiota primarily modulates metabolic pathways to adapt to the diverse nutrient environment within the host intestine. At the secondary level, the top ten pathways with the highest predicted functional abundance (Figure 10A) included global and overview maps, carbohydrate metabolism, amino acid metabolism, energy metabolism, translation, metabolism of cofactors and vitamins, membrane transport, and replication and repair. Among these, genes associated with carbohydrate metabolism exhibited the highest abundance, followed by those involved in amino acid metabolism. Carbohydrates and amino acids play crucial roles in microbial metabolism, serving as key substrates for energy acquisition by gut microbiota. Concurrently, microbial metabolic products, such as carbohydrates and amino acids, can be effectively absorbed and utilized by the host intestine. At the tertiary level, the predominant predicted functional pathways included metabolic pathways and biosynthesis of secondary metabolites (Figure 10B). Comparative analysis indicated that gut microbiota in both Sichuan-Tibetan black pigs and Landrace pigs exhibited a pronounced predominance of metabolic functions, consistent with their respective intestinal environments. Specifically, the gut microbiota of Sichuan-Tibetan black pigs was primarily enriched in pathways related to biosynthesis of secondary metabolites, microbial metabolism in diverse environments, and amino acid biosynthesis. In contrast, Landrace pigs showed higher enrichment in carbon metabolism and energy metabolism. Furthermore, the abundance of gut microbiota involved in the respective metabolic pathways was significantly higher in each breed compared to the other.

## 4. Discussion

The gut microbiota plays a crucial role in regulating host metabolism, immune function, and intestinal physiology, thereby significantly contributing to overall host health [13]. As a key mediator of host physiological processes, the complexity and plasticity of the porcine gut microbiota have become a major focus—and challenge—in modern animal husbandry and biomedical research. It is well established that different pig breeds exhibit distinct gut microbial structures, reflecting genetic, physiological, and adaptive differences. In this study, we applied metagenomic principles and high-throughput 16S rRNA sequencing to systematically compare the diversity, composition, and functional potential of the gut microbiota between Sichuan-Tibetan black pigs and Landrace pigs [16]. Notably, Xiao et al. [17] reported that age affects gut microbiota composition in Tibetan pigs (with higher alpha diversity in adults than in 4-month-old individuals and enriched Prevotella in juveniles), and such age-related variations have been documented across multiple pig breeds. However, this study controlled for age as a confounding factor by selecting pigs of the same age, so age did not impact the current results.

Our sequencing results revealed a total of 963 operational taxonomic units (OTUs) in Sichuan-Tibetan black pigs and 910 OTUs in Landrace pigs, collectively spanning 17 phyla, 99 families, 58 orders, 253 genera, and 457 species. Alpha diversity analysis indicated no significant differences in the Shannon, Simpson, or ACE indices between the two breeds; however, the Chao index differed significantly (*p* < 0.05). It should be clarified that the Chao index specifically reflects microbial community richness (the total number of species present), while the Shannon index accounts for both richness and evenness (the distribution uniformity of species abundance). The significant difference in the Chao index suggests that Landrace pigs have higher microbial richness compared to Sichuan-Tibetan black pigs. Beta diversity assessment via principal coordinate analysis (PCoA) and non-metric multidimensional scaling (NMDS) further demonstrated that while the microbial communities were structurally similar at the phylum level, they diverged significantly at the genus level. These findings also support Li et al. [18], who showed the relationship between pig breeds and gut microbiota structure and found that the gut microbiota composition of different breeds of pigs varies to some extent. This confirms the rationality of our conclusions.

Taxonomic composition analysis identified 17 phyla across both groups, including 16 core phyla and one phylum that was unique to Sichuan-Tibetan black pigs. The dominant phyla in both breeds were Firmicutes, Bacteroidetes, and Proteobacteria. However, Sichuan-Tibetan black pigs exhibited a significantly higher relative abundance of Firmicutes (73% vs. 62%) and a lower abundance of Bacteroidetes (20% vs. 29%) compared with Landrace pigs, with no marked differences in other phyla.

Functional prediction using BugBase and PICRUSt2 revealed significant phenotypic and metabolic distinctions between the two breeds. Specifically, the gut microbiota of Sichuan-Tibetan black pigs scored higher in stress tolerance, Gram-positive and Gram-negative composition, biofilm formation, and oxygen utilization—results supported by BugBase phenotype prediction (Figure 9) showing significant inter-breed differences (*p* < 0.05) in these phenotypic traits. KEGG pathway analysis indicated that metabolic processes dominated the functional profiles in both groups. This conclusion is consistent with the high stress resistance and high feed utilization rate of Sichuan-Tibetan black pigs to the environment, as well as the short slaughter cycle and strong immune ability of Landrace pigs [3]. It should be noted that Sichuan-Tibetan black pigs’ stress resistance and nutrient utilization efficiency, and Landrace pigs’ strong immune responses, are distinct adaptive traits mediated by different microbial functional pathways: the former is associated with secondary metabolite biosynthesis and diverse environmental metabolism, while the latter may relate to specific immune-modulating microbial taxa enriched in Landrace pigs. The results of this study are consistent with the previous research conclusions of Bai et al. [19] and Zeng et al. [20] on the composition of gut microbiota in Sichuan-Tibetan black pigs and Landrace pigs. At the same time, in-depth exploration was conducted on the structural differences and functions of gut microbiota between Sichuan-Tibetan black pigs and Landrace pigs. At finer metabolic levels, Sichuan-Tibetan black pigs were enriched in pathways related to carbohydrate metabolism and biosynthesis of secondary metabolites, whereas Landrace pigs showed greater enrichment in carbon and energy metabolism. These functional differences align with the known physiological traits of the two breeds: Sichuan-Tibetan black pigs exhibit stronger environmental resilience and feed efficiency, while Landrace pigs are characterized by rapid growth and robust immune capacity. A related study is very similar to the results of this study. Liu et al. [21] found that Tibetan pigs have rich and unique microbial composition and functional pathways. The phylum Verrucomicrobia and genus Akkermansia, which are related to immunity and disease resistance, are high-abundance bacterial taxa unique to the intestinal tract of Tibetan pigs, which may be related to their adaptation to high-altitude, low-oxygen, and low-temperature environments, and their disease resistance.

## 5. Conclusions

To elucidate the dynamic functional changes in gut microbiota, we applied 16S rRNA sequencing to analyze fresh fecal samples collected from Sichuan-Tibetan black pigs and Landrace pigs [22]. The results revealed that, in diversity assessments, only the Chao index exhibited a significant difference. Principal coordinate analysis indicated that while the microbial community structures of the two groups were highly similar at the phylum level, they differed significantly at the genus level. In terms of species composition, a total of 17 phyla were identified, 16 of which were core phyla. Notably, Sichuan-Tibetan black pigs harbored one unique phylum, whereas no endemic phylum was detected in Landrace pigs. The dominant phyla in both breeds were Firmicutes, Bacteroidetes, and Proteobacteria. However, Sichuan-Tibetan black pigs showed a higher abundance of Firmicutes and a lower abundance of Bacteroidetes, with minimal differences observed in other phyla. Phenotypic and functional predictions via BugBase indicated that the gut microbiota of Sichuan-Tibetan black pigs scored higher in five phenotypes, including stress tolerance. Furthermore, PICRUSt2 predictions revealed that the primary KEGG metabolic pathway was predominantly “metabolism.” At secondary and tertiary levels, Sichuan-Tibetan black pigs exhibited greater abundance in carbohydrate and secondary biomass metabolism, while Landrace pigs showed advantages in carbon and energy metabolism.

In summary, this study utilized high-throughput sequencing technology to analyze the gut microbiota of Sichuan-Tibetan black pigs and Landrace piglets, aiming to further explore structural differences and predict functional profiles of their gut microbial communities [23]. These findings help clarify the structural and functional variations in gut microbiota between the two pig breeds and provide a foundation for subsequent research on gut microbiota in related swine populations. And they also provide crucial metagenomic insights that can inform future strategies in genetic breeding and health management for swine, while also laying a theoretical foundation for optimizing husbandry practices and developing novel nutritional interventions from an ecological perspective.

## Data Availability

The raw 16S rRNA gene sequencing data have been deposited in the NCBI BioProject database under accession number PRJNA1302369.
